# Effects of changes in arterial pressure on organ perfusion during septic shock

**DOI:** 10.1186/cc10462

**Published:** 2011-09-21

**Authors:** Aurélie Thooft, Raphaël Favory, Diamantino Ribeiro Salgado, Fabio S Taccone, Katia Donadello, Daniel De Backer, Jacques Creteur, Jean-Louis Vincent

**Affiliations:** 1 Department of Intensive Care, Erasme Hospital, Université Libre de Bruxelles, Route de Lennik 808, 1070-Brussels, Belgium

## Abstract

**Introduction:**

Septic shock is characterized by altered tissue perfusion associated with persistent arterial hypotension. Vasopressor therapy is generally required to restore organ perfusion but the optimal mean arterial pressure (MAP) that should be targeted is uncertain. The aim of this study was to assess the effects of increasing MAP using norepinephrine (NE) on hemodynamic and metabolic variables and on microvascular reactivity in patients with septic shock.

**Methods:**

This was a single center, prospective, interventional study conducted in the medico-surgical intensive care unit of a university hospital. Thirteen patients in septic shock for less than 48 hours who required NE administration were included. NE doses were adjusted to obtain MAPs of 65, 75, 85 and (back to) 65 mmHg. In addition to hemodynamic and metabolic variables, we measured thenar muscle oxygen saturation (StO_2_), using near infrared spectroscopy (NIRS), with serial vaso-occlusive tests (VOTs) on the upper arm. We also evaluated the sublingual microcirculation using sidestream dark field (SDF) imaging in 6 of the patients.

**Results:**

Increasing NE dose was associated with an increase in cardiac output (from 6.1 to 6.7 l/min, *P*<0.05) and mixed venous oxygen saturation (SvO_2_, from 70.6 to 75.9%, *P*<0.05). Oxygen consumption (VO_2_) remained stable, but blood lactate levels decreased. There was a significant increase in the ascending slope of StO_2 _(from 111 to 177%/min, *P*<0.05) after VOTs. SDF imaging showed an increase in perfused vessel density (PVD, from 11.0 to 13.2 n/mm, *P*<0.05) and in microvascular flow index (MFI, from 2.4 to 2.9, *P*<0.05).

**Conclusions:**

In this series of patients with septic shock, increasing MAP above 65 mmHg with NE was associated with increased cardiac output, improved microvascular function, and decreased blood lactate concentrations. The microvascular response varied among patients suggesting that individualization of blood pressure targets may be warranted.

## Introduction

Septic shock is characterized by an alteration in tissue perfusion associated with persistent arterial hypotension - generally defined as a systolic arterial pressure of less than 90 mm Hg [1] - despite adequate fluid resuscitation [2]. This leads to organ dysfunction and even death in around 50% of cases [3]. Evaluation of systemic hemodynamic variables can be inadequate to identify tissue perfusion, which is directly influenced by additional microvascular factors. De Backer and colleagues [4] showed that sepsis is associated with reduced microvascular density, an increased number of non-perfused small vessels, and heterogeneity among microcirculatory areas. There are several possible reasons for these abnormalities, including the release of mediators and cytotoxic substances - such as free oxygen radicals, various cytokines, and prostanoids - which can alter endothelial function and cell deformability, induce vascular hyporeactivity to catecholamines [5], induce coagulation abnormalities [6], increase cell adhesion to endothelial cells, and induce interstitial edema. Because of its vasoactive effects, norepinephrine (NE) could contribute to alter the microcirculation and impair cellular metabolism.

In septic shock, fluid resuscitation is insufficient to restore hemodynamic stability, so that vasopressor therapy is typically required to restore organ perfusion. Recommendations suggest that a mean arterial pressure (MAP) of around 65 mm Hg should initially be targeted [1]. There is some suggestion that an MAP below this value may be associated with a worse evolution [7]. An arterial pressure that is too low induces a loss of autoregulation of organ flow, so that tissue perfusion becomes directly dependent on the arterial pressure level. However, whether a higher MAP should be targeted is a matter of debate. Ledoux and colleagues [8] showed that increasing MAP from 65 to 75 and to 85 mm Hg did not influence urinary output, blood lactate levels, or gastric intramucosal partial pressure of carbon dioxide (PCO_2_) in 10 patients with septic shock. Bourgoin and colleagues [9] observed that increasing MAP from 65 to 85 mm Hg did not modify creatinine clearance or blood lactate level. However, Deruddre and colleagues [10] showed that, although renal perfusion evaluated by Doppler ultrasonography did not change overall when MAP was increased above 65 mm Hg, individual response was highly variable and several patients did have a marked increase in renal perfusion. Jhanji and colleagues [11] showed that increasing MAP from 60 to 70, 80, and 90 mm Hg by increasing the NE dose could increase oxygen delivery (DO_2_) and cutaneous microvascular flow; however, the authors found no significant effect on the microcirculation when using sidestream dark field (SDF) imaging techniques. Using the same SDF imaging techniques and a similar protocol with MAP at 65, 75, and 85 mm Hg, Dubin and colleagues [12] reported no change in the sublingual microcirculation. All of these data suggest that considerable interpatient variability can occur and that the optimal MAP level remains unclear.

Because of these unsettled controversies, we studied the hemodynamic, metabolic, and microcirculatory effects of an increase in NE dose in patients with septic shock. Our hypothesis was that increasing MAP by increasing NE doses would improve microvascular function in patients with septic shock.

## Materials and methods

This was a single-center study conducted over a 1-year period and approved by the local ethics committee of Erasme University Hospital (Brussels, Belgium). Informed consent was obtained from all patients or their closest relative.

### Patients

We studied 13 adult patients who had had septic shock for less than 48 hours. All patients had an arterial catheter, a central venous catheter, and a pulmonary artery catheter (Swan-Ganz) in place. All patients required an NE infusion for arterial hypotension resistant to fluid therapy, supported by a change in pulse pressure (ΔPP) of less than 13% [13]. All patients were treated with mechanical ventilation and received an infusion of midazolam for sedation and morphine for analgesia. Inotropic support was provided with dobutamine when judged necessary by the attending physician. The patients were all stabilized and had undergone initial resuscitation. Exclusion criteria were need for an MAP of higher than 65 mm Hg (for example, severe head trauma, recent stroke, or advanced arteriopathy), Child-Pugh C cirrhosis, scleroderma or drepanocytosis, significant arrhythmias, pregnancy, or refusal to participate in the study.

### Protocol

NE doses were adjusted to maintain a baseline MAP of 65 ± 2 mm Hg. After stabilization, basal measurements were performed twice, 15 minutes apart ('baseline 1 and 2'), and the NE doses were increased to obtain an MAP of 75 ± 2 mm Hg; patients were allowed to stabilize for 30 minutes before taking measurements ('increasing 1'). NE doses were then increased to reach an MAP of 85 ± 2 mm Hg within 15 minutes, and the measurements were repeated after another 30-minute stabilization period ('increasing 2'). NE doses were then decreased to return to an MAP of 65 ± 2 mm Hg, and another stabilization period of 30 minutes was allowed before the final measurements ('baseline 3'). During the protocol, no other change in treatment was allowed.

### Measurements

We measured temperature, heart rate, cardiac output, systemic and pulmonary arterial pressures, central venous pressure (CVP), pulmonary artery occlusion pressure, and ΔPP. Arterial and venous gases were obtained to determine pH, arterial partial pressure of carbon dioxide (PaCO_2_), arterial partial pressure of oxygen (PaO_2_), hemoglobin concentration, arterial hemoglobin saturation (SaO_2_), mixed venous hemoglobin saturation (SvO_2_), and blood lactate concentration. Vascular resistances were calculated by standard formulas. The Acute Physiology and Chronic Health Evaluation II (APACHE II) score [14] and the Sequential Organ Failure Assessment (SOFA) score [15] were calculated.

In each patient, we used near-infrared spectroscopy (NIRS) (InSpectra™ Model 650; Hutchinson Technology Inc., Hutchinson, MN, USA) [16,17] to evaluate tissue oxygenation non-invasively by using the differences in absorption of near-infrared light by oxyhemoglobin and deoxyhemoglobin. The NIRS probe was placed on the thenar eminence to measure tissue hemoglobin oxygen saturation (StO_2_). A vascular occlusion test (VOT) [18] was used to measure different variables reflecting local metabolic demand and microcirculatory function. The VOT was performed by inflating a pneumatic cuff around the upper arm to 50 mm Hg above the systolic pressure for 3 minutes to follow the evolution of StO_2 _and global tissue hemoglobin (THI). The descending slope of the StO_2 _and the nirVO_2_I (the reverse descending slope multiplied by the average THI during the first occlusion minute) [19] estimate regional tissue oxygen consumption (VO_2_). The ascending slope of the StO_2 _and the reactive hyperemia reflect microcirculatory reactivity (that is, the vessels' capacity to adjust oxygen extraction).

In six patients, we also assessed the sublingual microcirculation using the SDF imaging technique [20] (Microscan, Microvision Medical, Amsterdam, The Netherlands), in which a green light is reflected by the background and absorbed by hemoglobin in red blood cells flowing in superficial vessels. These six patients had the same characteristics as the others. We based our analysis on previously established criteria for microcirculatory perfusion [21] by obtaining 20-second video clips of five different sites by a device with a × 5 objective lens. The data were analyzed blindly and randomly. Using a cutoff diameter of 20 μm, we separated the vessels into two categories: small (corresponding to capillaries) and large (corresponding to venules). We also determined two perfusion indices: the microvascular flow index (MFI) and the vessel density. The MFI characterizes microvascular flow as absent (0), intermittent (1), sluggish (2), or normal (3). The functional capillary density (FCD) is estimated by the measurement of perfused vessel density (PVD) (that is, the proportion of perfused vessels, multiplied by vessel density).

### Statistical analysis

Data were analyzed using SPSS 13.0 for Windows (SPSS Inc., Chicago, IL, USA). Descriptive statistics were computed for all study variables. To verify the normality of the distributions of continuous variables, histograms and normal quantile plots were examined and the Kolmogorov-Smirnov test was used. Continuous variables are presented as mean (95% confidence intervals) if normally distributed and as median (25% to 75% interquartile range) if not normally distributed. Categorical variables are presented as number and percentage. To analyze the time courses of the hemodynamic, metabolic, or NIRS parameters, we used analysis of variance for repeated measurement followed by paired *t *test or Friedman test followed by the Wilcoxon test. To correct for multiple comparison, a Bonferroni correction was used. All statistics were two-tailed, and a *P *value of less than 0.05 was considered to be significant. After checking that there was no significant difference between the first two baseline measurements, we averaged them to obtain only one baseline.

## Results

Clinical data of the 13 patients are presented in Table 1. The most common source of sepsis was abdominal infection. The mean duration of shock prior to study inclusion was 24.1 ± 14.7 hours. Our patients had already undergone initial resuscitation and had been in shock for an average of 24 hours. Three patients received dobutamine (4 μg/minute). The mean baseline CVP was 12 mm Hg, cardiac index was 3.6 L/minute-m², SvO_2 _was 70%, and ΔPP was less than 13% (Table 2). NE doses during the study are shown in Table 2. There were no adverse effects associated with the increasing NE doses.

**Table 1:** Clinical data

Parameter	Value
Age in years, mean (95% CI)	63.3 (55.0-71.6)
Males/females	9/4
Weight in kilograms, mean (95% CI)	80 (69-91)
Body mass index, mean (95% CI)	27.3 (22.6-32.0)
APACHE II score, mean (95% CI)	22.7 (18.9-26.5)
SOFA score, mean (95% CI)	12.8 (11.4-14.2)
Source of infection, number (percentage)	
Abdominal	6 (46)
Lung	5 (39)
Unknown	2 (15)
Comorbidities, number (percentage)	
Chronic hypertension	8 (62)
Diabetes	5 (38)
Chronic renal failure	2 (15)
ICU length of stay in days, median (25-75% IQR)	17 (6-27)
28-day mortality, number (percentage)	2 (17)

APACHE II, Acute Physiology and Chronic Health Evaluation II; ICU, intensive care unit; SOFA, Sequential Organ Failure Assessment; CI: confidence interval; IQR: interquartile range

**Table 2:** Norepinephrine doses and hemodynamic variables

	65 mm Hg	75 mm Hg	85 mm Hg	65 mm Hg
Norepinephrine, μg/minute	17.3 (5.9-28.6)	24.5 (9.5-39.5)^a^	32.7 (14.0-51.4)^a^	22.4 (11.0-33.8)
Mean arterial pressure, mm Hg	66.0 (65.1-66.8)	75.9 (73.5-78.3)^a^	86.3 (84.3-88.4)^a^	66.2 (65.1-67.3)
Heart rate, beats per minute	93.5 (83.9-103.1)	91.4 (81.4-101.5)	92.8 (82.1-103.6)	94.2 (81.7-106.7)
Cardiac output, liters per minute	6.1 (5.4-6.8)	6.5 (5.7-7.3)	6.7 (5.9-7.6)^a^	6.1 (5.4-6.8)
Mean pulmonary arterial pressure, mm Hg	29 (24.9-33.1)	30.3 (26-34.7)	32.2 (27.9-36.5)^a^	29.4 (24.8-34.0)
Pulmonary artery occlusion pressure, mm Hg	15.3 (12.7-18)	15.4 (12.7-18.2)	16.1 (13.5-18.6)	14.6 (12.2-17.1)
Central venous pressure, mm Hg	12.0 (9.8-14.3)	12.8 (10.3-15.2)	13.4 (11.1-15.7)^a^	12.4 (10.3-14.5)
Systemic vascular resistance, dyne/second per cm^−5^	716 (647-786)	803 (711-896)^a^	902 (793-1,010)^a^	726 (645-806)
Pulmonary vascular resistance, dyne/second per cm^−5^	181 (141-222)	188 (141-234)	197 (150-244)	184 (134-234)
Change in pulse pressure, percentage	2 (1.5-4.5)	2.5 (1-8)	1.5 (0-4)	4 (3-7)

^a^*P *<0.05 versus baseline.

### Hemodynamic and metabolic variables

Cardiac output increased from 6.1 L/minute (5.4 to 6.8 L/minute) at baseline to 6.7 L/minute (5.9 to 7.6 L/minute) at an MAP of 85 mm Hg (*P *<0.05) without significant changes in heart rate (Tables 2 and 3). Systemic vascular resistance also increased. SvO_2 _increased from 70.6% (67.9% to 73.2%) to 75.9% (71.7% to 80.1%) (*P *<0.05). DO_2 _increased but VO_2 _remained stable. Blood lactate concentrations decreased.

**Table 3:** Metabolic variables

	65 mm Hg	75 mm Hg	85 mm Hg	65 mm Hg
Hemoglobin, g/dL	8.9 (8.0-9.7)	9 (8.2-9.9)	8.9 (7.9-10.0)	8.8 (7.8-9.8)
Temperature, °C	37.3 (36.5-38.0)	37.3 (36.5-38.1)	37.3 (36.6-38.1)	37.4 (36.6-38.2)
Lactate, mEq/L	2.3 (1.5-3.1)	2.2 (1.4-2.9)^a^	2.1 (1.4-2.8)^a^	2.2 (1.5-3.0)
SaO_2_, percentage	97.2 (96.4-98.1)	97.5 (96.7-98.3)	97.9 (96.9-98.9)	97.8 (96.9-98.7)
SvO_2_, percentage	70.6 (67.9-73.2)	72.3 (69.3-75.3)	75.9 (71.7-80.1)^a^	69.0 (65.8-72.2)
DO_2_, mL/minute	728 (633-824)	806 (678-933)	826 (687-965)^a^	721 (623-819)
VO_2_, mL/minute	197 (175-220)	203 (173-232)	177 (149-204)	208 (179-238)
EO_2_, mL/minute	27.5 (24.6-30.4)	25.8 (22.6-29.0)	22.5 (18.3-26.7)^a^	29.4 (26.1-32.7)

^a^*P *<0.05 versus baseline. DO_2_, oxygen delivery; EO_2_, oxygen extraction ratio; SaO_2_, arterial hemoglobin saturation; SvO_2_, mixed venous oxygen saturation; VO_2_, oxygen consumption.

### Near-infrared spectroscopy variables

**Figure 1:**
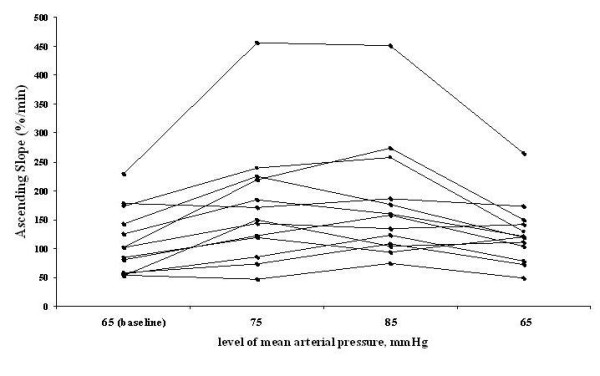
**Ascending slope in individual patients**. There was a systematic response of the slope to the increase in mean arterial pressure.

The ascending slope increased from 110.6%/minute (80.0 to 141.2%/minute) at baseline to 176.9%/minute (121.6 to 232.2%/minute) at 85 mm Hg (Figure 1) (Table 4). There was no significant change in the descending slope or in the nirVO_2_I.

**Table 4:** Near-infrared spectroscopy and sidestream dark field variables

	65 mm Hg	75 mm Hg	85 mm Hg	65 mm Hg
NIRS variables				
StO_2 _baseline, percentage	82.5 (79.0-85.9)	81.0 (77.0-85.0)	81.2 (77.6-84.7)	77.8 (73.6-82.1)
Descending slope, percentage/minute	−14.8 (−18.0 - −11.6)	−14.5 (−18.9 - −10.0)	−16.4 (−22.0 - −10.9)	−15.7 (−21.9 - −9.4)
nirVO_2_I, arbitrary units	139 (114-165)	122 (98-146)	153 (109-196)	129 (93-165)
Delta StO_2_, percentage	7.4 (5.3-9.5)	8.4 (5.2-11.6)	7.7 (5.4-9.9)	7.4 (5-9.7)
AUC ratio, percentage	0.11 (0.05-0.16)	0.12 (0.09-0.15)	0.10 (0.08-0.14)	0.10 (0.07-0.15)
Ascending slope, percentage/minute	111 (80-141)	172 (115-228)^a^	177 (122-232)^a^	114 (94-134)
SDF variables				
Total vessel density	12.8 (10.5-15.0)	13.2 (11.1-15.3)	14.3 (12.9-15.7)	13.7 (11.0-16.4)
Small vessel density	10.7 (8.5-12.9)	11 (8.5-13.4)	11.6 (10.1-13.1)	11.7 (9.0-14.3)
PVD, vessels/mm	11.0 (8.6-13.3)	12.0 (9.4-14.5)	13.2 (11.9-14.5)^a^	12.1 (9.2-14.9)
Small PVD, vessels/mm	9.1 (6.4-11.9)	9.8 (6.9-12.8)	10.7 (9.0-12.3)	10.3 (7.1-13.5)
All PPV, percentage	85.9 (80.1-91.6)	89.5 (83.7-95.2)	92.6 (89.9-95.3)	87.7 (79.9-95.6)
Large PPV, percentage	95.5 (93.6-97.4)	95.4 (88.8-102.0)	97.5 (95.2-99.7)	89.8 (82.7-96.9)
Small PPV, percentage	83.6 (76.1-91.0)	87.9 (81.8-94.0)	91.1 (87.9-94.3)	86.4 (76.3-96.5)
Microvascular flow index	2.4 (2.2-2.7)	2.7 (2.4-2.9)	2.9 (2.8-2.9)^a^	2.5 (2.2-2.9)

^a^*P *<0.05 versus baseline. AUC, area under the curve; NIRS, near-infrared spectroscopy; nirVO_2_I, reverse descending slope multiplied by the average tissue hemoglobin index during the first occlusion minute; PPV, proportion of perfused vessels; PVD, perfused vessel density; SDF, sidestream dark field; StO_2_, thenar muscle oxygen saturation.

### Sidestream dark field variables

**Figure 2:**
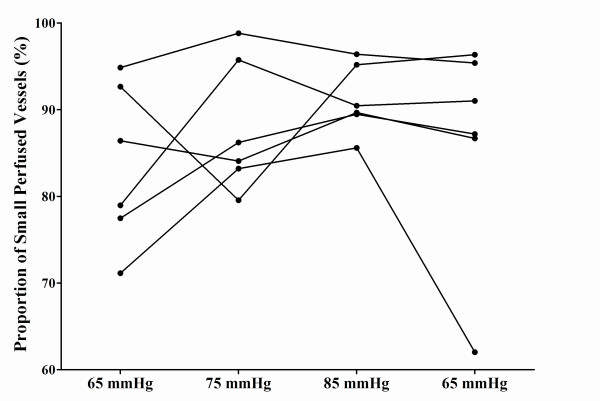
**Proportion of small perfused vessels in individual patients**.

**Figure 3:**
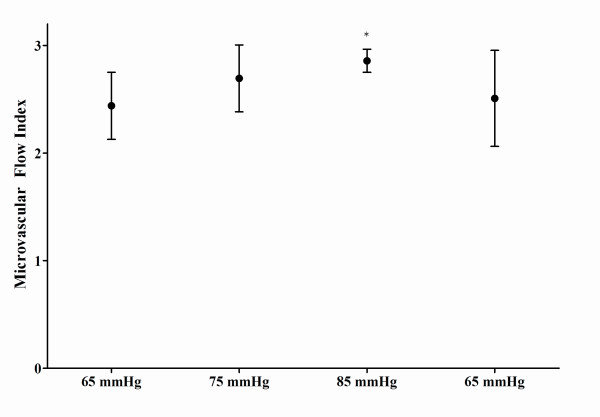
**Microvascular flow index**. Data are presented as median (interquartile range). *Significant difference at 5% level (Bonferroni correction) versus baseline.

There was considerable interindividual variability in the proportion of small perfused vessels (Figure 2 and Table 4). Global PVD showed a significant increase from 11.0 vessels per millimeter (8.6 to 13.3 vessels per millimeter) to 13.2 vessels per millimeter (11.9 to 14.5 vessels per millimeter), but the differences were not significant for small vessels. The MFI showed variable values between patients but was mainly sluggish at baseline (2.4, 2.2 to 2.7) and increased significantly toward a normal flow (2.9, 2.8 to 2.9) when MAP increased to higher levels (Figure 3).

## Discussion

The major finding of this study was that increasing MAP from 65 to 75 and 85 mm Hg by titrating NE doses was associated with an increase in cardiac output, a decrease in blood lactate level, and an improvement in microcirculatory function as evaluated by NIRS. The study of the microcirculation by SDF in six patients showed some improvements characterized by an increase in perfused vessel density and a normalization of blood flow in these vessels.

Our patients had already undergone initial resuscitation and had been in shock for an average of 24 hours. To ensure that alterations in the variables were not influenced by factors other than the MAP increase associated with NE, no other changes in treatment were allowed during the study period. Moreover, there was no temperature change during the short observation period. To rule out the possible influence of the natural course of the shock, one last measurement was made when the MAP had returned to the baseline level of 65 mm Hg.

NE is a commonly used vasopressor agent in septic shock. Its strong alpha-adrenergic properties make it a very effective vasopressor agent [22], and it is now considered the vasopressor of choice [23]. Nevertheless, questions arise as to whether this vasoconstriction is not deleterious for the microcirculation. Increasing the doses of NE was associated with increases in cardiac output, DO_2_, and SvO_2_. Interestingly, in this situation of sepsis, which is characterized by relative cardiac depression, the increase in cardiac output could be explained by the beta-1 adrenergic action of NE. Although there was no significant increase in VO_2 _in our study, blood lactate concentrations decreased slightly. This suggests that tissue blood flow and oxygen supply were increased without modification of global VO_2_. Vessel blood flow depends on, among other factors, the diameter of the vessel and the applied pressure gradient. The vasoconstricting effects of NE were manifest by a significant increase in systemic vascular resistance; however, the increase in cardiac output may have contributed to the increased microvascular flow observed in our study.

The microvascular changes were modified in parallel with an increase in cardiac output and in systemic vascular resistance. As all variables move in the same direction, we can hypothesize that both these variables were involved in the modifications but there were no correlations between the changes in the ascending slope and the increase in cardiac output or in systemic vascular resistance. As this is probably a situation of loss of arterial pressure autoregulation, regional microvascular changes (like recruitment) may partly explain the significant differences.

Microcirculatory alterations have been demonstrated in sepsis [4] and are particularly marked in non-survivors [18,24], improving in those patients who survive but persisting in patients who develop organ failure. These microvascular alterations are largely related to endothelial cell dysfunction and loss of the ability to regulate vascular tone [25]. The study of vascular reactivity with NIRS - using the occlusive test - provides an estimation of microvessel function [18]. This hypoxic stimulus promotes the 'recruitment', namely the opening, of non-perfused capillaries, followed by so-called 'reactive hyperemia'. With NIRS, impairment in tissue reperfusion after hypoxia in the thenar eminence has been shown to be correlated with the severity of sepsis and with outcome [18].

StO_2 _was not affected by the different arterial pressure levels and remained lower than in a healthy population [19]. The absence of changes in StO_2 _suggests that NIRS changes were not the consequence of the increase in cardiac output. The ascending slope of the StO_2_, the delta between StO_2 _at baseline and at the hyperemia peak, and the ratio between the two areas under the curves can be used to estimate capillary reactivity. In our study, although delta StO_2 _and the ratio between the areas under the curves did not change, the StO_2 _reperfusion slope increased when the arterial pressure was increased by NE, indicating some improvement in microvascular reactivity. To compare our variables with values found in other NIRS studies during sepsis, we can express the StO_2 _recovery slope results in percentage per second [18,19]. The ascending slope at baseline was around 1.9%/second, which is markedly altered and corresponds to values observed in septic shock [19]. When MAP was increased to 85 mm Hg, the ascending slope increased to 3.1%/second, a value seen in healthy volunteers. In Figure 1, we can see variability in the reperfusion slope, indicating that some patients derived greater benefit from this intervention than did others. The tissue VO_2 _can be estimated from either the descending StO_2 _slope - which shows the rate of tissue VO_2 _at the onset of hypoxia - or the nirVO_2_I [19], which corresponds to a decrease in StO_2 _during the first minute of hypoxia weighted by the conversion rate of oxyhemoglobin into deoxyhemoglobin during this period. None of these variables changed, indicating that increasing NE dose has no effect on the local tissue VO_2 _despite the microvascular recruiting reflected by the rise in StO_2_.

Our study also showed an improvement in microvascular reactivity, evaluated by NIRS, related to the changes in arterial pressure during sepsis. As expected, there was some interpatient variability. Our data are in line with the observation that correction of severe hypotension (MAP of less than 65 mm Hg) by NE administration in patients with septic shock resulted in improvement in microvascular reactivity measured by NIRS [26]. It is, of course, difficult to totally rule out a pharmacologic effect of NE on the ascending slope but the concordant changes in sublingual microcirculation suggest that it was related to improved microvascular perfusion.

We also used SDF techniques to study the sublingual microcirculation in 6 of the 13 patients. There was an increase in the proportion of all perfused vessels and of perfused small vessels. The changes were not significant, but these parameters evolved from abnormal values at baseline - as expected in patients with sepsis - to almost normal values when the MAP was increased to 85 mm Hg. Almost all large vessels were perfused, and this is expected when the probe is properly applied without too much local pressure (this is actually a quality control measure). Although the changes in small PVD were not significant, there was a significant increase in the global PVD (that is, some improvement in FCD), which probably can be explained by the increase in the proportion of small perfused vessels. Finally, the MFI showed variable values between patients but was mainly sluggish at baseline and increased toward a normal flow at higher MAP values. Accordingly, increasing arterial pressure above 65 mm Hg in septic shock may have beneficial effects on perfusion and flow in the microvessels in patients with septic shock.

Our results contradict those of some previous studies [8,9,11], but several factors may explain these differences. For example, Ledoux and colleagues [8] used dopamine in addition to NE, and the use of a second vasoactive agent may have influenced the vascular reactivity. Bourgoin and colleagues [9] observed significant increases in cardiac index, SvO_2_, and DO_2 _but no significant changes in renal perfusion. It should be noted that their period of observation was quite long. Moreover, these data are in contradiction to the work of Deruddre and colleagues [10], who reported a significant increase in urinary output in a shorter study period. Two other studies [11,12] used SDF to assess microcirculatory changes after increasing MAP by NE during sepsis and found no significant changes in microcirculatory variables. The apparent differences between these studies and the present study may be related to differences in fluid repletion or in the timing of the study or both. In the study by Jhanji and colleagues [11], three patients needed fluid administration, suggesting that resuscitation may not have been complete. Moreover, the inclusion time varied from 12 hours to 3.5 days after the onset of septic shock, likely with different microcirculation reactivity among patients. Finally, patient selection can also represent an important explanation for the differences with the study of Dubin and colleagues [12], in which an important variability in the response was observed among the patients, some of whom presented an improvement in microvascular perfusion during the increase in NE dosage.

Our study has several limitations. First, we included a relatively small number of patients, as did other studies [8-12], and had strict enrollment criteria. We excluded those who died early despite initial resuscitation attempts and those who could not be stabilized, and patients with notable comorbidities (like cirrhosis, arteriopathy, or impaired cardiac function) were also excluded. Moreover, our patients all had normal or high cardiac output. Therefore, extrapolation of our results to all patients in septic shock and notably unstable patients or those with hypodynamic shock may be problematic. In addition, the SDF measurements were not performed on all patients. Second, we did not include a control group. However, the patients were used as their own control, as we obtained measurements before and after manipulation of blood pressure. Since there was no difference between the final measurements and the first baseline level, we can exclude an influence of spontaneous changes over time. Finally, this study investigated only immediate changes to the increase in arterial pressure caused by increased doses of NE. To extrapolate the impact of these changes to periods in which the MAP is maintained above 65 mm Hg for longer would be difficult, namely because of the likely influence of concurrent events. More fundamentally, interindividual differences are likely so that having an identical MAP target for all patients is probably naïve. In addition, these data cannot be extrapolated to other vasopressor agents, and although we observed no adverse effects associated with the increasing dose of NE in our study, higher doses of NE over longer periods may be associated with a greater risk of potential adverse events, including arrhythmias.

## Conclusions

In the treatment of patients in septic shock, increasing MAP above 65 mm Hg with higher doses of NE can result in increased cardiac output, improved microcirculatory function, and decreased lactate concentrations. The present observations challenge the commonly heard statement that an MAP of 65 to 70 mm Hg is adequate for most patients with septic shock and suggest that microvascular studies may be helpful to define the optimal target in a given individual. The microvascular response is variable between patients and suggests that personal adaptation of the MAP is needed. Additional studies should be carried out to develop criteria for determining an individually adapted arterial pressure threshold and to evaluate the long-term effects of this strategy.

## Key messages

• Increasing mean arterial pressure by norepinephrine during septic shock can increase cardiac output and improve microvascular flow and reactivity in stable resuscitated patients without modification of global oxygen consumption.

• There is considerable interindividual variability in microvascular response, suggesting that the level of mean arterial pressure should be adapted to each patient.

## Abbreviations

CVP: central venous pressure; DO_2_: oxygen delivery; FCD: functional capillary density; MAP: mean arterial pressure; MFI: microvascular flow index; NE: norepinephrine; NIRS: near-infrared spectroscopy; nirVO_2_I: reverse descending slope multiplied by the average tissue hemoglobin index during the first occlusion minute; PP: pulse pressure; PVD: perfused vessel density; SDF: sidestream dark field; StO_2_: thenar muscle oxygen saturation; SvO_2_: mixed venous oxygen saturation; THI: tissue hemoglobin index; VO_2_: oxygen consumption; VOT: vascular occlusion test.

## Competing interests

The authors declare that they have no competing interests.

## Authors' contributions

AT participated in the design of the study and helped conduct the study, collect the data, perform the microcirculatory imaging and the statistical analyses, and draft the manuscript. DDB participated in the design of the study and helped perform the statistical analyses and draft the manuscript. JC and J-LV participated in the design of the study. RF, DRS, and KD helped conduct the study, collect the data, and perform the microcirculatory imaging. FST helped conduct the study, collect the data, perform the microcirculatory imaging and the statistical analyses, and draft the manuscript. All authors read and approved the final manuscript.

## References

[B1] Dellinger RP Levy MM Carlet JM Bion J Parker MM Jaeschke R Reinhart K Angus DC Brun-Buisson C Beale R Calandra T Dhainaut JF Gerlach H Harvey M Marini JJ Marshall J Ranieri M Ramsay G Sevransky J Thompson BT Townsend S Vender JS Zimmerman JL Vincent JL Surviving Sepsis Campaign: international guidelines for management of severe sepsis and septic shock: 2008 Crit Care Med 2008 36 296 327 10.1097/01.CCM.0000298158.12101.41 18158437

[B2] Levy MM Fink MP Marshall JC Abraham E Angus D Cook D Cohen J Opal SM Vincent JL Ramsay G 2001 SCCM/ESICM/ACCP/ATS/SIS International Sepsis Definitions Conference Crit Care Med 2003 31 1250 1256 10.1097/01.CCM.0000050454.01978.3B 12682500

[B3] Vincent JL Sakr Y Sprung CL Ranieri VM Reinhart K Gerlach H Moreno R Carlet J Le Gall JR Payen D Sepsis in European intensive care units: results of the SOAP study Crit Care Med 2006 34 344 353 10.1097/01.CCM.0000194725.48928.3A 16424713

[B4] De Backer D Creteur J Preiser JC Dubois MJ Vincent JL Microvascular blood flow is altered in patients with sepsis Am J Respir Crit Care Med 2002 166 98 104 10.1164/rccm.200109-016OC 12091178

[B5] Landin L Lorente JA Renes E Canas P Jorge P Liste D Inhibition of nitric oxide synthesis improves the vasoconstrictive effect of noradrenaline in sepsis Chest 1994 106 250 256 10.1378/chest.106.1.250 8020279

[B6] Leclerc J Pu Q Corseaux D Haddad E Decoene C Bordet R Six I Jude B Vallet B A single endotoxin injection in the rabbit causes prolonged blood vessel dysfunction and a procoagulant state Crit Care Med 2000 28 3672 3678 10.1097/00003246-200011000-00023 11098972

[B7] Varpula M Tallgren M Saukkonen K Voipio-Pulkki LM Pettila V Hemodynamic variables related to outcome in septic shock Intensive Care Med 2005 31 1066 1071 10.1007/s00134-005-2688-z 15973520

[B8] Ledoux D Astiz ME Carpati CM Rackow EC Effects of perfusion pressure on tissue perfusion in septic shock Crit Care Med 2000 28 2729 2732 10.1097/00003246-200008000-00007 10966242

[B9] Bourgoin A Leone M Delmas A Garnier F Albanese J Martin C Increasing mean arterial pressure in patients with septic shock: effects on oxygen variables and renal function Crit Care Med 2005 33 780 786 10.1097/01.CCM.0000157788.20591.23 15818105

[B10] Deruddre S Cheisson G Mazoit JX Vicaut E Benhamou D Duranteau J Renal arterial resistance in septic shock: effects of increasing mean arterial pressure with norepinephrine on the renal resistive index assessed with Doppler ultrasonography Intensive Care Med 2007 33 1557 1562 10.1007/s00134-007-0665-4 17486316

[B11] Jhanji S Stirling S Patel N Hinds CJ Pearse RM The effect of increasing doses of norepinephrine on tissue oxygenation and microvascular flow in patients with septic shock Crit Care Med 2009 37 1961 1966 10.1097/CCM.0b013e3181a00a1c 19384212

[B12] Dubin A Pozo MO Casabella CA Palizas F Jr Murias G Moseinco MC Kanoore E Palizas F Estenssoro E Ince C Increasing arterial blood pressure with norepinephrine does not improve microcirculatory blood flow: a prospective study Crit Care 2009 13 R92 10.1186/cc7922 19534818 PMC2717464

[B13] Michard F Boussat S Chemla D Anguel N Mercat A Lecarpentier Y Richard C Pinsky MR Teboul JL Relation between respiratory changes in arterial pulse pressure and fluid responsiveness in septic patients with acute circulatory failure Am J Respir Crit Care Med 2000 162 134 138 10903232 10.1164/ajrccm.162.1.9903035

[B14] Knaus WA Draper EA Wagner DP Zimmerman JE APACHE II: a severity of disease classification system Crit Care Med 1985 13 818 829 10.1097/00003246-198510000-00009 3928249

[B15] Vincent JL Moreno R Takala J Willatts S De Mendonca A Bruining H Reinhart CK Suter PM Thijs LG The SOFA (Sepsis-related Organ Failure Assessment) score to describe organ dysfunction/failure. On behalf of the Working Group on Sepsis-Related Problems of the European Society of Intensive Care Medicine Intensive Care Med 1996 22 707 710 10.1007/BF01709751 8844239

[B16] Mancini DM Bolinger L Li H Kendrick K Chance B Wilson JR Validation of near-infrared spectroscopy in humans J Appl Physiol 1994 77 2740 2747 7896615 10.1152/jappl.1994.77.6.2740

[B17] Lima A Bakker J Noninvasive monitoring of peripheral perfusion Intensive Care Med 2005 31 1316 1326 10.1007/s00134-005-2790-2 16170543

[B18] Creteur J Carollo T Soldati G Buchele G De Backer D Vincent JL The prognostic value of muscle StO2 in septic patients Intensive Care Med 2007 33 1549 1556 10.1007/s00134-007-0739-3 17572876

[B19] Skarda DE Mulier KE Myers DE Taylor JH Beilman GJ Dynamic near-infrared spectroscopy measurements in patients with severe sepsis Shock 2007 27 348 353 10.1097/01.shk.0000239779.25775.e4 17414414

[B20] Ince C The microcirculation is the motor of sepsis Crit Care 2005 9 Suppl 4 S13 S19 10.1186/cc3753 16168069 PMC3226164

[B21] De Backer D Hollenberg S Boerma C Goedhart P Buchele G Ospina-Tascon G Dobbe I Ince C How to evaluate the microcirculation: report of a round table conference Crit Care 2007 11 R101 10.1186/cc6118 17845716 PMC2556744

[B22] Martin C Papazian L Perrin G Saux P Gouin F Norepinephrine or dopamine for the treatment of hyperdynamic septic shock? Chest 1993 103 1826 1831 10.1378/chest.103.6.1826 8404107

[B23] De Backer D Biston P Devriendt J Madl C Chochrad D Aldecoa C Brasseur A Defrance P Gottignies P Vincent JL Comparison of dopamine and norepinephrine in the treatment of shock N Engl J Med 2010 362 779 789 10.1056/NEJMoa0907118 20200382

[B24] Sakr Y Dubois MJ De Backer D Creteur J Vincent JL Persistent microcirculatory alterations are associated with organ failure and death in patients with septic shock Crit Care Med 2004 32 1825 1831 10.1097/01.CCM.0000138558.16257.3F 15343008

[B25] Vallet B Endothelial cell dysfunction and abnormal tissue perfusion Crit Care Med 2002 30 S229 S234 10.1097/00003246-200205001-00010 12004241

[B26] Georger JF Hamzaoui O Chaari A Maizel J Richard C Teboul JL Restoring arterial pressure with norepinephrine improves muscle tissue oxygenation assessed by near-infrared spectroscopy in severely hypotensive septic patients Intensive Care Med 2010 36 1882 1889 10.1007/s00134-010-2013-3 20689910

